# Association of blood lead with estradiol and sex hormone-binding globulin in 8-19-year-old children and adolescents

**DOI:** 10.3389/fendo.2023.1096659

**Published:** 2023-02-08

**Authors:** Kaiyu Pan, Rongliang Tu, Zixiu Cai, Yingdan Huang, Chengyue Zhang

**Affiliations:** ^1^Department of Paediatrics, The First People’s Hospital of Xiaoshan District, Hangzhou, Zhejiang, China; ^2^Department of Neonatology, Zhejiang Xiaoshan Hospital, Hangzhou, Zhejiang, China; ^3^Department of Paediatrics, The Second People's Hospital of Xiaoshan District, Hangzhou, Zhejiang, China; ^4^Xiangya School of Medicine, Central South University, Changsha, Hunan, China

**Keywords:** blood lead, estradiol, SHBG, children, adolescents

## Abstract

**Background:**

Metals can interfere with hormonal functioning through indirect mechanisms and by binding at the receptor site; thus, they may be associated with hormonal changes. However, there have been few studies on the health impact of metal exposure among children and adolescents. Thus, we aimed to examine the associations of blood lead level (BLL) with estradiol (E2) and sex hormone-binding globulin (SHBG) among children and adolescents aged 8–19 years in the National Health and Nutrition Examination Survey (NHANES) database.

**Methods:**

This was a cohort study of 2188 individuals from the NHANES. BLL was taken as independent variables, E2 and SHBG as dependent variable. We conducted weighted multivariate linear regression models and smooth curve fittings to evaluate the association between them.

**Results:**

The BLL was significantly positively associated with serum SHBG level in females, especially when the LnBLL quartiles are between Q3 and Q4. There was an inverted U-shaped association between BLL and E2 with the point of inflection at 1.86 μg/L and a U-shaped association between BLL and SHBG with the point of inflection at 1.86 μg/L in female adolescents aged 16-19 years. Meanwhile, In males, there was a positive trend of correlation between BLL and E2 in the 8-11 years, and 16-19 years groups.

**Conclusions:**

This study found an inverted U-shaped association of BLL with E2 and a U-shaped association between BLL and SHBG in female adolescents aged 16-19 years. This indicates that adjusting blood lead exposure to mitigate the effects of lead on growth and development is important for adolescents aged 16-19 years. Controlling the BLL below 1.86 μg/L may minimize the damage to E2.

## 1 Introduction

Estrogen (E) plays an important role in hormonal regulation in women (1). It maintains and promotes female secondary sexual characteristics and the function of gonads and serves as an important biomarker for the onset of puberty, menstrual status, and fertility (2). There are three types of estrogens: estrone (E1), estradiol (E2), and estriol (E3). Of these, E2 is the most physiologically relevant and has the highest affinity for estrogen receptors ERα and ERβ (3). In particular, E2 is directly related to menarche and helps breast maturation in puberty (4, 5). In males, E2 is also essential to reproductive function, body composition, and glucose metabolism (6). Furthermore, an experiment in female mice showed that E2 as a pubertal hormone is essential for the maturation of the frontal cortex, which regulates many cerebral functions, including executive functions, language, temporal integration, emotional behavior, and working memory (7). One study showed that E2 had a positive association with total testosterone (TT) in males, which is the most important male reproductive hormone (8).

Sex hormone-binding globulin (SHBG) is produced in the liver. As a transport carrier that binds E2 and regulates its biological activity, SHBG is thought to reflect metabolic levels and regulate the plasma levels and bioavailability of E2 (9, 10). E2 is tightly bound to SHBG in the circulation, and only a small free fraction is considered biologically active (11). E2 and SHBG have been shown to be influenced by various factors, such as insulin, thyroid hormone, and environmental endocrine disruptors, in which heavy metals like lead play an important role (12–14). Poisoning caused by lead, one of the ten most harmful metals listed by the World Health Organization (WHO), is a serious public health concern (15). Blood lead level (BLL) can be a homeostatic marker of lead exposure (16).

Lead exposure is unavoidable, and its sources include drinking water, contaminated soil particles, chemical industry, and mining (17–19). Among children and adolescents, lead exposure even at low levels can induce neuropsychological deficits in cognition, attention, behavior, intelligence, and memory (20). Blood lead has E receptor activity and has endocrine-disrupting properties even at low concentrations (21). Some animal experiment and child studies have shown that lead exposure may influence pubertal development through the hypothalamic–pituitary–gonadal axis (22, 23). In addition, children and adolescents are at the age when they are most sensitive to the adverse health effects of endocrine disruptors (24). Further, serum E2 and SHBG levels in puberty children are closely associated with growth and development. Thus, understanding the mechanism by which blood lead influences variations in E2 and SHBG levels may provide additional insight and opportunities for prevention lead exposure. However, there are few correlational studies on the effects of lead exposure on reproductive hormones in children and adolescents aged 8-19 years. Thus, the present study used BLL as the independent variable and E2 and SHBG as the dependent variables to determine the association of blood lead with E2 and SHBG in the general population of 8-19-year-old children and adolescents in the United States. Further, we explored the possible mechanisms of action of this association to provide evidence for avoiding and preventing diseases (e.g. delayed menarche, impaired gonadal development) and improving child-related health worldwide.

## 2 Materials and methods

### 2.1 Data source and study population

Data were collected from National Health and Nutrition Examination Surveys (NHANES), a large data registry of health and nutritional status of nationally representative samples in the United States. Information is available online (http://www.cdc.gov/nchs/nhanes.htm).

**Figure 1:**
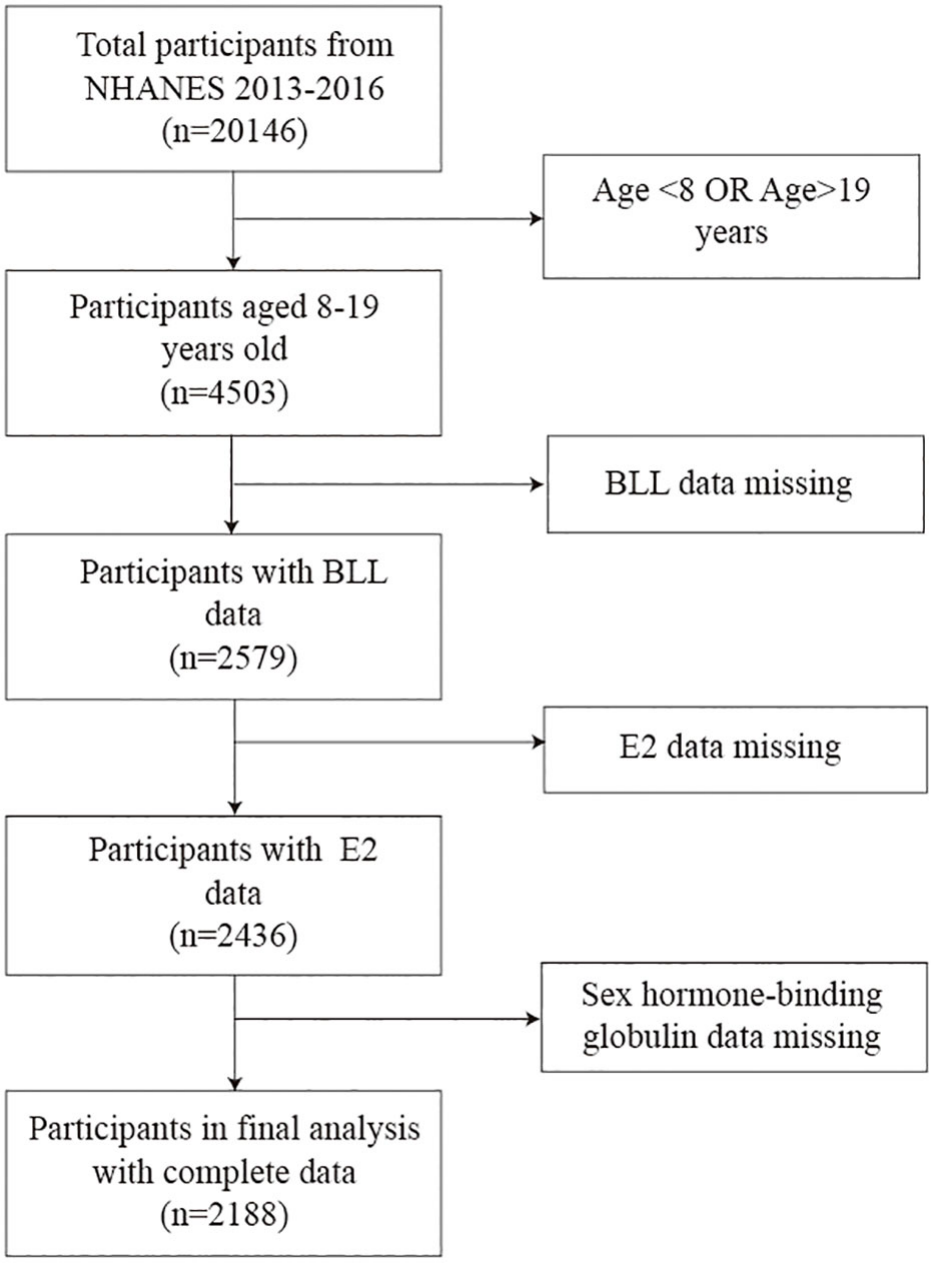
Flow diagram illustrating the process of participant selection. BLL, Blood lead levels; E2, Estradiol; SHBG, Sex hormone-binding globulin.

According to the guidelines published by the WHO in 2017, adolescents have been most recently defined as those aged 10-19 years. In this study, we selected children and adolescents aged 8-19 years in whom E2 and SHBG are the most associated with growth and development, as the study population (25). Moreover, we selected these two data cycles (2013-2014, 2015-2016) because the data on E2 for children and adolescents aged 8-19 years were only available for these two periods in data from 2011 to 2020. We screened 20146 participants from NHANES 2013-2016; of them, 4503 children and adolescents aged 8-19 years were included. After excluding those with missing BLL (n=1924), E2 (n=143), and SHBG (n=248) data, we finally enrolled 2188 eligible participants. The participant selection process is shown in **Figure 1**. The home interviews assessed the family and sample person demographics and other aspects at home. Adolescents aged over 16 years and emancipated minors were interviewed directly, while participants aged <16 years and those unable to answer questions themselves were provided with information by proxy.

All surveys have obtained National Center for Health Statistics (NCHS) Research Ethics Review Board (ERB) approval. The protocol numbers are available at https://www.cdc.gov/nchs/nhanes/irba98.htm, as accessed on January 4, 2023.

### 2.2 E2, SHBG, and BLL measurements

The level of serum E2 (all unconjugated, including free and protein-bound forms) was measured by quantitative analysis using isotope dilution high-performance liquid chromatography tandem mass spectrometry with stable isotope labeled internal standards and external calibrators (https://wwwn.cdc.gov/nchs/data/nhanes/2015-2016/labmethods/TST_I_MET_TST_EST.pdf). Meanwhile, the level of SHBG (bound and free types) in human serum and plasma matricesl was determined using the test based on the reaction of SHBG with immuno-antibodies and chemo-luminescence measurements of the corresponding reaction products (https://wwwn.cdc.gov/nchs/data/nhanes/2015-2016/labmethods/TST_I_MET_SHBG.pdf).

For BLL measurement, whole blood samples were stored frozen (-30°C) and then shipped to National Center for Environmental Health for testing. The BLL was quantitatively determined using inductively coupled plasma-dynamic reaction cell-mass spectrometry (https://wwwn.cdc.gov/nchs/data/nhanes/2015-2016/labmethods/PBCD_I_met.pdf).

### 2.3 Covariates

Covariates are variables that can affect the regression coefficient of the exposure variable by more than 10% when introduced into the basic model or excluded from the full model. Based on previous studies (20, 21), we finally selected the following variables as possible confounders on the relationship between exposure and outcome variables: age, sex, race/ethnicity, ratio of family income to poverty; total energy, cholesterol, iron, and zinc intake on the first day, fish eaten during the past 30 days; moderate recreational activities; body mass index; haematocrit, serum cotinine; serum albumin, serum copper, and serum zinc. BLL is negatively correlated with HCT, and HCT-adjusted whole blood lead may be a better biomarker of lead hematotoxicity than the unadjusted BLL (26). As the primary metabolites of nicotine, serum cotinine can be used as markers for active smoking and as indices for secondhand smoke exposure, which is important in children and adolescents who are often passive recipients of tobacco. Dietary cholesterol was included because serum cholesterol may affect the binding of SHBG to E2. In addition, metal intake and data related to serum metal levels were also analyzed as several metals are known to have an association with reproductive hormones. Detailed interview and laboratory procedures of the above covariates are available on the website.

### 2.4 Statistical analysis

Considering the presence of missing values for covariates such as serum copper, serum zinc, and albumin, we used multiple interpolation to reduce the possible bias caused by missing values and the loss of test efficacy. Multiple linear regression analysis was performed on each of the five sets of random data formed by multiple interpolation, and the regression coefficients and standard errors of the five regression models were combined and tabulated. To investigate the effects of different levels of confounders, we constructed three multiple linear regression models as follows: Model 1 was the unadjusted model; model 2 was adjusted for the confounders of age, sex, and race/ethnicity as the minimally adjusted model; and model 3 was adjusted for all confounders associated with them as the fully adjusted model. Given the left-skewed distribution of blood lead, serum estradiol, and SHBG among the 8-19-year-old participants, they were ln-transformed to LnBLL, LnE2, and LnSHBG, respectively. Accordingly, serum copper, zinc, and albumin; total iron and zinc intake on the first day all underwent the same manipulation.

For the comparison of baseline data in different blood lead quartile groups, we used weighted linear regression models to calculate continuous variables and a weighted chi-square test to calculate categorical variables. Besides, multiple linear regression equations were used to explore the relationship between LnBLL and LnE2 or between LnBLL and LnSHBG, with three linear regression models for continuous variables (details of adjustment of confounders for each model in **Supplementary Tables 1****,** **2**) and chi-square tests for categorical variables achieving stratified analyses and *post hoc* subgroup comparisons. Furthermore, trend tests were performed for the LnBLL quartile grouping to enhance the strength of the evidence and the sensitivity of the test, and the results were considered positive when *P* for trend was also significant.

For the nonlinear relationship between the independent and dependent variables, we used threshold effect analysis to find the inflection points of the quantitative-effective relationship curves between LnBLL and LnE2, or LnBLL and LnSHBG in different age groups (27, 28). Therefore, we divided the participants into three age groups: 8 to 11 year-old age group, 12 to 15 year-old age group and 16 to 19 year-old age group. They consisted of 1152 people (576 males and 576 females), 550 people (269 males and 281 females), and 486 people (238 males and 248 females), respectively. The fitted model with the largest likelihood value was found by recursive experimental method, and the results were considered significant if the *P*-value was <0.05 in the log-likelihood ratio test, which indicated that there was an inflection point in the smooth fitting curve. Two-piecewise linear regression was then performed to further test the turning effect (27). All statistical analyses were performed using the Empower software (www.empowerstats.com; X&Y solutions, Boston MA), R version 3.4.3 (http://www.R-project.org, R Foundation) with weighted processing.

## 3 Results

### 3.1 Participant characteristics

There were significant differences in age, sex, race/ethnicity, ratio of family income to poverty, moderate recreational activities; total energy, iron, and zinc intake on the first day; fish eaten during the past 30 days; body mass index; hematocrit; serum cotinine; serum copper; serum zinc; estradiol; and SHBG between the different blood lead quartile subgroups. The results of the weighted characterization of the study population are presented in **Table 1**.

**Table 1:** Characteristics and statistical description of children and adolescent participants aged 8-19 in NHANES 2013–2016 (n=2188).

	Blood lead level (μmol/L)					*P*value
	Total	Q1 (0.002~0.016)	Q2 (0.017~0.024)	Q3 (0.025~0.035)	Q4 (>0.036)	
Age (years)	12.7 ± 3.4	13.4 ± 3.2	12.8 ± 3.4	12.3 ± 3.3	12.2 ± 3.6	<0.0001
8 to 11 years old	9.5 ± 1.1	9.7 ± 1.1	9.5 ± 1.1	9.6 ± 1.2	9.3 ± 1.1	<0.0001
12 to 15 years old	13.5 ± 1.1	13.7 ± 1.1	13.5 ± 1.1	13.5 ± 1.0	13.2 ± 1.1	<0.0001
16 to 19 years old	17.3 ± 1.1	17.1 ± 1.1	17.4 ± 1.1	17.4 ± 1.1	17.6 ± 1.2	<0.0001
Sex (%)						<0.0001
Male	50.2	33.7	43.7	58.2	67.3	
Female	49.8	66.3	56.3	41.8	32.7	
Race/ethnicity (%)						<0.0001
Non-Hispanic White	53.4	52.7	56.6	51.7	51.7	
Non-Hispanic Black	12.2	8.4	9.2	14.9	16.9	
Mexican American	16.3	22.0	15.1	16.4	12.2	
Other race/ethnicity	18.1	16.9	19.1	17.0	19.2	
Ratio of family income to poverty	2.3 ± 1.5	2.6 ± 1.6	2.5 ± 1.5	2.3 ± 1.5	1.9 ± 1.3	<0.0001
Moderate recreational activities (%)						<0.0001
Yes	28.9	27.0	34.7	29.0	23.2	
No	24.3	35.4	21.4	18.6	22.5	
Not recorded	46.8	37.7	43.9	52.4	54.3	
Total nutrient intake on the first day–Energy (kcal)	2017.7 ± 882.2	1985.5 ± 856.9	1965.2 ± 780.9	2019.7 ± 942.5	2117.5 ± 955.4	0.0248
Total nutrient intake on the first day–Cholesterol (mg)	241.3 ± 199.0	246.7 ± 214.5	237.9 ± 179.9	228.1 ± 209.4	254.0 ± 193.9	0.1776
Total nutrient intake on the first day–Iron (mg)	14.9 ± 9.5	15.0 ± 9.3	14.3 ± 8.8	14.6 ± 8.3	16.0 ± 11.6	0.0169
Total nutrient intake on the first day–Zinc (mg)	10.8 ± 7.7	10.7 ± 6.8	10.4 ± 6.9	10.4 ± 6.6	11.9 ± 10.1	0.0032
Fish eaten during the past 30 days (%)						<0.0001
Yes	40.4	38.4	38.0	39.9	46.1	
No	59.4	52.2	55.8	50.3	40.1	
Not recorded	9.2	9.3	6.2	9.7	13.8	
Body mass index (kg/m^2^)	22.1 ± 5.9	23.5 ± 6.7	22.5 ± 6.0	21.5 ± 5.3	20.7 ± 5.2	<0.0001
Hematocrit (%)	40.5 ± 3.3	39.9 ± 3.1	40.7 ± 3.3	40.6 ± 3.4	40.7 ± 3.4	0.0001
Serum cotinine (ng/mL)	5.8 ± 37.9	1.6 ± 15.5	3.7 ± 32.0	5.1 ± 30.0	13.5 ± 60.8	<0.0001
Serum albumin (g/L)	45.2 ± 3.0	45.0 ± 2.8	45.3 ± 3.1	44.9 ± 2.9	45.6 ± 2.9	0.0599
Serum copper (umol/L)	17.3 ± 4.0	18.1 ± 4.6	17.3 ± 3.6	16.8 ± 3.9	17.0 ± 3.7	0.0011
Serum zinc (umol/L)	12.5 ± 2.2	12.2 ± 2.1	12.8 ± 2.2	12.7 ± 2.4	12.4 ± 2.2	0.0238
Estradiol (pg/mL)	31.5 ± 54.4	47.7 ± 73.5	34.0 ± 51.1	23.8 ± 39.9	19.9 ± 43.0	<0.0001
SHBG (nmol/L)	69.1 ± 47.3	68.2 ± 55.4	63.7 ± 41.0	69.6 ± 44.9	76.6 ± 47.5	0.0001

Mean ± SD for continuous variables like age, ratio of family income to poverty; total energy, cholesterol, iron and zinc intake on the first day; body mass index, hematocrit, serum continine, serum albumin, serum copper, serum zinc, estradiol, SHBG. P value was calculated by weighted linear regression model.

% for Categorical variables like race/ethnicity, moderate recreational activities, fish eaten during the past 30 days. P value was calculated by weighted chi-square test.

SHBG, Sex hormone-binding globulin.

### 3.2 Association of LnBLL with LnE2 and LnBLL with LnSHBG

**Figure 2:**
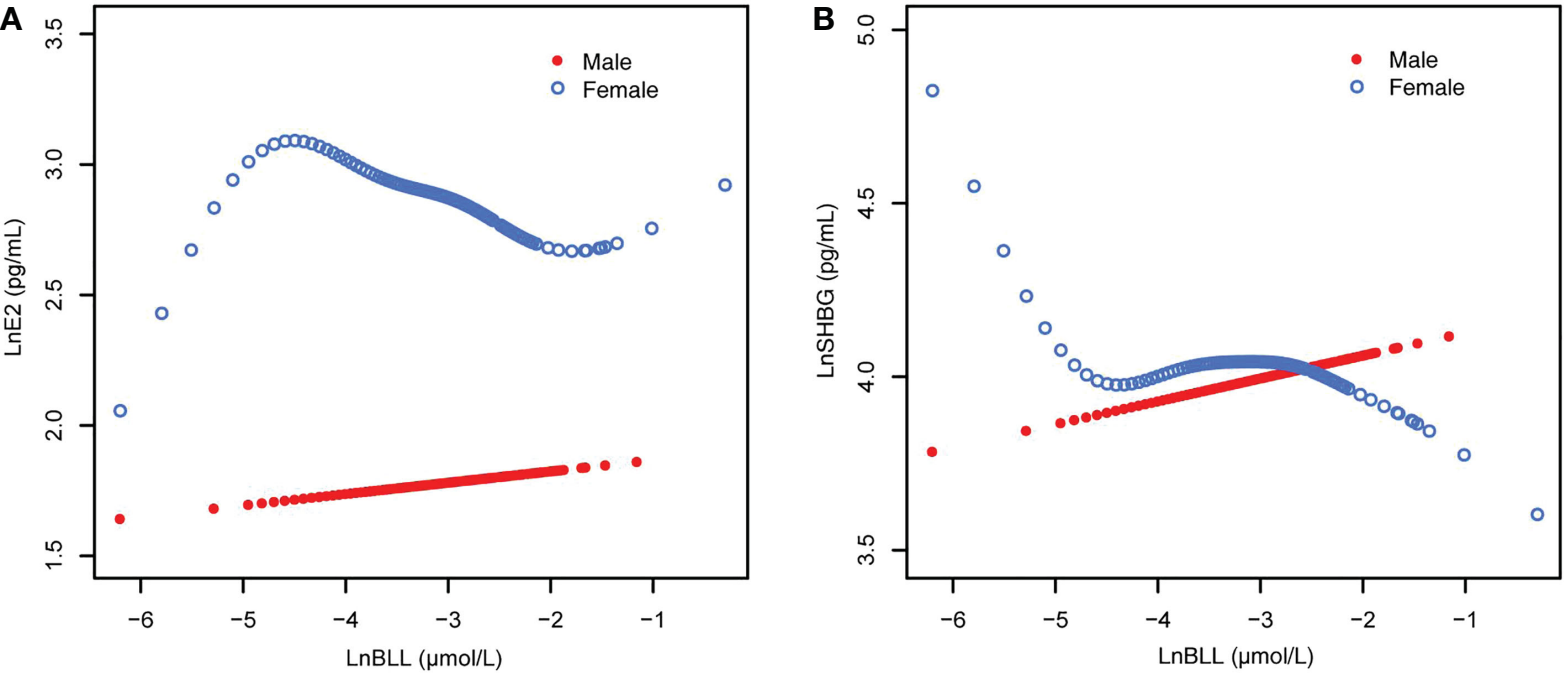
The associations of BLL with E2 and SHBG, stratified by age. **(A)** BLL and E2 dose–response relationship. Adjusted for age, ratio of family income to poverty; total energy, cholesterol, Ln(iron) and Ln(zinc) intake on the first day; fish eaten during the past 30 days, moderate recreational activities, body mass index and Ln(serum copper). **(B)** BLL and SHBG dose–response relationship. Adjusted for age, ratio of family income to poverty; total energy, Ln(iron) and Ln(zinc) intake on the first day; fish eaten during the past 30 days, moderate recreational activities, body mass index, hematocrit, serum continine, Ln(serum albumin), Ln(serum copper) and Ln(serum zinc). BLL, Blood lead levels; E2, Estradiol; SHBG, Sex hormone-binding globulin.

Overall, the relationship between BLL and E2 is detailed in **Supplementary Table 1**, and results by age group are presented in **Table 2**. After stratifying the data by race/ethnicity, we found a significant negative association between BLL and E2 only among non-Hispanic Black and Mexican American participants in the unadjusted model (Model 1). However, no significant association was found after adjusting for covariates (Models 2 and 3). After sex stratification, there was a significant trend of varying LnE2 in males and females for each increase in LnBLL (*P* for trend <0.05) in model 3. This association was positive in males and negative in females (**Figure 2**) and was especially significant in females (*P* for trend <0.001 in all three models).

**Table 2:** Association between LnBLL (μmol/L) and LnE2 (pg/mL) Stratified by race/ethnicity and sex.

LnBLL (μmol/L) (Quartile)	LnE2 (pg/mL)		
	8 to 11 years old	12 to 15 years old	16 to 19 years old
Male			
Q1	Reference	Reference	Reference
Q2	0.0868 (0.0460, 0.1277) ^***^	1.7147 (0.4118, 3.0176) ^*^	0.2173 (0.1510, 0.2837) ^***^
Q3	0.0430 (0.0037, 0.0823) ^*^	3.0914 (1.7980, 4.3847) ^***^	0.1553 (0.0871, 0.2235) ^***^
Q4	0.1073 (0.0673, 0.1473) ^***^	1.6038 (0.2687, 2.9388) ^*^	0.1560 (0.0906, 0.2215) ^***^
*P* for trend	<0.001	0.326	0.006
Female			
Q1	Reference	Reference	Reference
Q2	-0.0788 (-0.1760, 0.0183)	-3.3109 (-11.8286, 5.2069)	0.0795 (-0.0585, 0.2175)
Q3	-0.0756 (-0.1791, 0.0278)	-16.4467 (-27.4173, -5.4762) ^**^	-0.0019 (-0.1705, 0.1668)
Q4	-0.0632 (-0.1739, 0.0476)	-3.1587 (-16.2987, 9.9813)	-0.1939 (-0.3980, 0.0102)
*P* for trend	0.308	0.377	0.171

In this chart, age, ratio of family income to poverty; total energy, cholesterol, Ln(iron) and Ln(zinc) intake on the first day; fish eaten during the past 30 days, moderate recreational activities, body mass index, Ln(serum copper) were adjusted.

BLL, Blood lead levels; E2, Estradiol.

^*^P  < 0.05, ^**^P  < 0.01, ^***^P  < 0.001.

**Figure 3:**
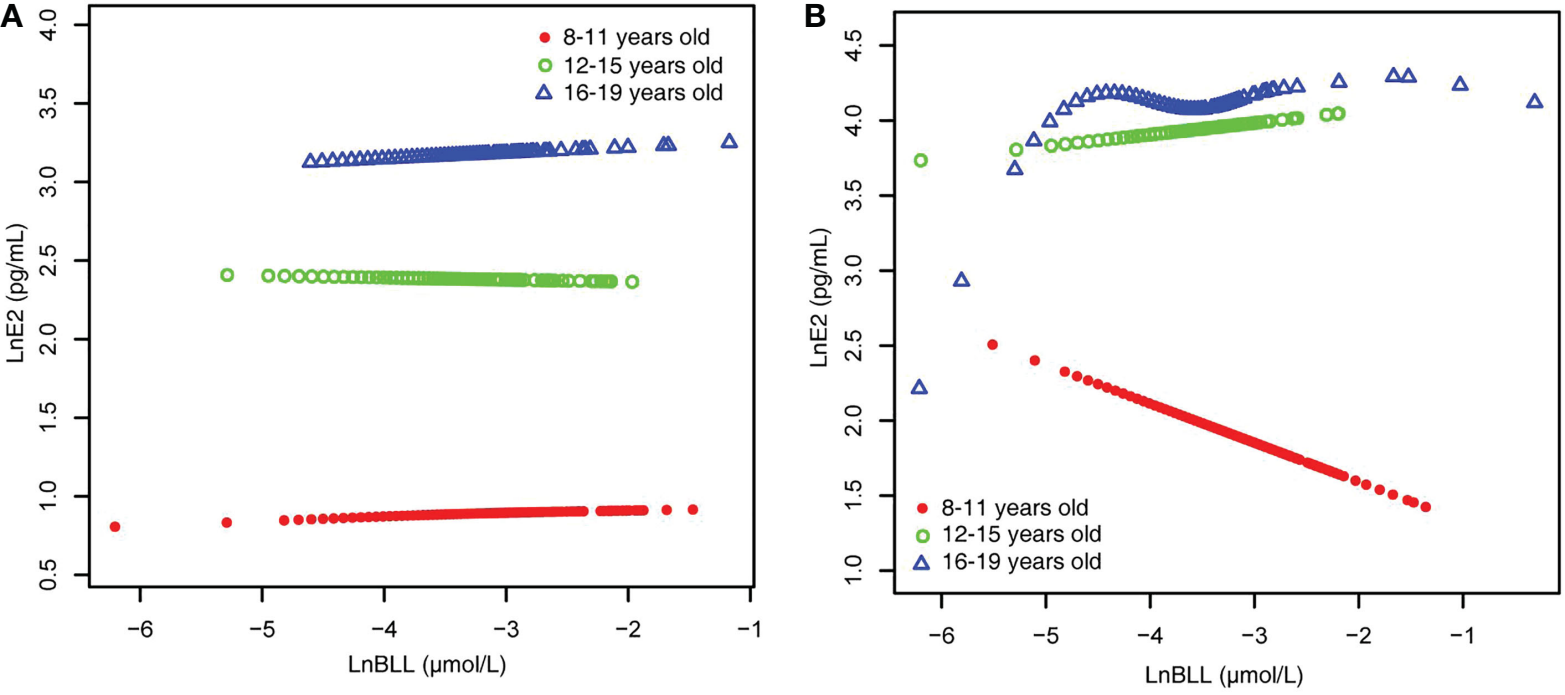
BLL and E2 dose–response relationship, stratified by age. **(A)** male. **(B)** female. Adjusted for ratio of family income to poverty; total energy, cholesterol, Ln(iron) and Ln(zinc) intake on the first day; fish eaten during the past 30 days, moderate recreational activities, body mass index and Ln(serum copper). BLL, Blood lead levels; E2, Estradiol.

When stratified by age and adjusted for all covariates, we found an increase in LnE2 to varying degrees with LnBLL quartiles between Q2 and Q4 for male participants in all age groups compared with that in the lowest quartile group of LnBLL. There was a significant correlation between LnBLL and LnE2, especially a significant linear trend in the 8-11-year-old group (*P* for trend <0.001) and 16-19-year-old group (*P* for trend=0.006) (**Table 2**). In contrast, the association did not reach statistical significance for linear correlation among female children and adolescents in each of the three age groups. Smoothed curve fit plots (**Figure 3**) stratified by age supported the results.The multiple linear regression models for the association of BLL with SHBG are shown in **Supplementary Table 2**, as well as the results by age group in **Table 3**. BLL and SHBG were positively associated in model 2 (adjusted for sex, age, and race/ethnicity) (*P <*0.05), while no significant association was observed in neither model 1 (unadjusted model) nor 3 (fully adjusted model). After stratification by race/ethnicity, the association between BLL and SHBG was significantly positive among non-Hispanic Black and Mexican American populations. However, after adjusting for all covariates, this association was not observed across races. The results after sex stratification showed a significant relationship between LnBLL and LnSHBG for both sexes in each model (*P* for trend <0.001), indicating that LnSHBG tended to significantly increase with every quartile increase in LnBLL. Further, we found a more significantly positive correlation in female participants with LnBLL quartiles between Q3 and Q4, demonstrating a significant difference in all three models.

**Table 3:** Association between LnBLL (μmol/L) and LnSHBG (pg/mL) Stratified by race/ethnicity and sex.

LnBLL (μmol/L) (Quartile)	LnSHBG (pg/mL)		
	8 to 11 years old	12 to 15 years old	16 to 19 years old
Male			
Q1	Reference	Reference	Reference
Q2	-0.0035 (-0.0497, 0.0427)	-0.0311 (-0.1016, 0.0394)	-0.1099 (-0.1755, -0.0444) ^**^
Q3	-0.0331 (-0.0774, 0.0113)	0.0559 (-0.0147, 0.1264)	-0.0458 (-0.1133, 0.0216)
Q4	0.0510 (0.0055, 0.0965) ^*^	0.0806 (0.0084, 0.1527) ^*^	0.0694 (0.0049, 0.1340) ^*^
*P* for trend	0.030	0.001	<0.001
Female			
Q1	Reference	Reference	Reference
Q2	0.0002 (-0.0415, 0.0419)	0.0266 (-0.0344, 0.0877)	0.0019 (-0.0689, 0.0728)
Q3	0.0665 (0.0224, 0.1106) ^**^	-0.0124 (-0.0913, 0.0665)	0.2767 (0.1895, 0.3640) ^***^
Q4	0.0467 (-0.0011, 0.0945)	0.1327 (0.0400, 0.2255) ^**^	0.2638 (0.1596, 0.3681) ^***^
*P* for trend	0.003	0.051	<0.001

In this chart, age, ratio of family income to poverty; total energy, Ln(iron) and Ln(zinc) intake on the first day; fish eaten during the past 30 days, moderate recreational activities, body mass index, hematocrit, serum continine, Ln(serum albumin), Ln(serum copper), Ln(serum zinc) were adjusted.

BLL, Blood lead levels; SHBG, Sex hormone-binding globulin.

^*^P  < 0.05, ^**^P  < 0.01, ^***^P  < 0.001.

**Figure 4:**
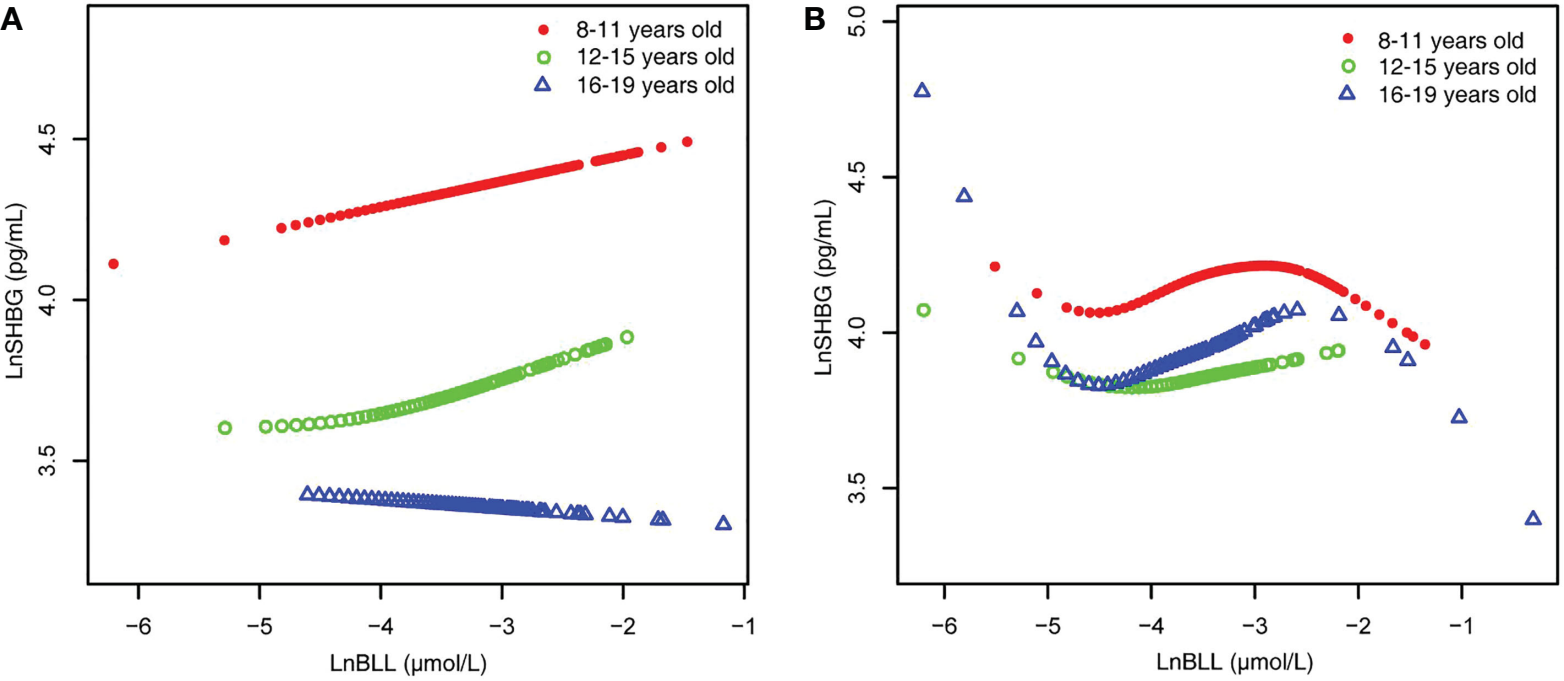
BLL and SHBG dose–response relationship, stratified by age. **(A)** male. **(B)** female. Adjusted for ratio of family income to poverty; total energy, Ln(iron) and Ln(zinc) intake on the first day; fish eaten during the past 30 days, moderate recreational activities, body mass index, hematocrit, serum continine, Ln(serum albumin), Ln(serum copper) and Ln(serum zinc). BLL, Blood lead levels; SHBG, Sex hormone-binding globulin.

When stratified by age and sex (**Table 3**), we found that LnBLL and LnSHBG were correlated in male children and adolescents of all ages, with significant results as seen in trend tests; particularly, LnSHBG significantly increased with LnBLL in the Q4 range compared to LnSHBG in the lowest LnBLL quartile group (8-11-year-old group, 0.0510 [0.0055, 0.0965]; 12-15-year-old group, 0.0806 [0.0084, 0.1527]; and 16-19-year-old group, 0.0694 [0.0049, 0.1340]). Moreover, among male adolescents aged 16-19 years, we found a decrease in LnSHBG in the group with LnBLL of Q2 compared to that in group with LnBLL of Q1. Meanwhile, the relationship between LnBLL and LnSHBG was significant in female participants aged 8-11 years and 16-19 years. When LnBLL were between Q3 (0.2767 [0.1895, 0.3640]) and Q4 (0.2638 [0.1596, 0.3681]), LnSHBG showed a more prominent increase in female adolescents aged 16-19 years compared to LnSHBG with LnBLL quartiles in Q1. Smoothed curve fit plots (**Figure 4**) stratified by age supported these results.

Because the smoothed curve fit plots (**Figure 4**) after age stratification showed nonlinear relationships between LnBLL and LnE2 and between LnBLL and LnSHBG, we implemented threshold effects analysis and segmented linear regression models to fit the data and find meaningful inflection points in the dose-response curves of the independent and dependent variables for male and female children and adolescents of different ages. Among female participants aged 16-19 years, a significant difference between the two segmented regression coefficients was found for LnBLL and LnE2 before and after -4.7 μmol/L, and the log-likelihood ratio test was = 0.029<0.05, showing that LnBLL increased with LnE2 in adolescents aged 16-19 years before LnBLL was -4.7 μmol/L. In contrast, opposite effects were observed after -4.7 μmol/L (**Table 4**; **Figure 3**). An inverted U-shaped curve association was found. Similarly, there was a turning point in the LnBLL and LnSHBG dose-response relationship curve in female adolescents aged 16-19 years (log-likelihood ratio test <0.001), showing a U-shaped curve (**Table 4**; **Figure 4**). Beyond that, the LnBLL and LnE2 relationship curve in female participants aged 16-19 years in the remaining zones and the LnBLL and LnSHBG relationship curve in male participants aged 12-15 years, female participants aged 12-15 years, and female participants aged 16-19 years (in the remaining zones) all presented a linear relationship (*P* > 0.05).

**Table 4:** Threshold effect analysis and two-piecewise linear regression of LnBLL (μmol/L) on LnE2 (pg/mL) or LnSHBG (pg/mL).

	LnE2	LnSHBG	Adjusted ß (95% CI), *P*-value
Female			
16-19 years old	LnBLL <-4.7 (μmol/L)		1.1845 (0.0525, 2.3164) 0.0414
	LnBLL >-4.7 (μmol/L)		-0.1306 (-0.3898, 0.1286) 0.3243
		LnBLL <-4.7 (μmol/L)	-0.8556 (-1.4203, -0.2909) 0.0033
		LnBLL >-4.7 (μmol/L)	0.1945 (0.0644, 0.3245) 0.0037

For two-piecewise linear regression model of LnBLL on LnE2, age, sex, race/ethnicity, ratio of family income to poverty; total energy, cholesterol, Ln(iron) and Ln(zinc) intake on the first day; fish eaten during the past 30 days, moderate recreational activities, body mass index, Ln(serum copper) were adjusted.

For two-piecewise linear regression model of LnBLL on LnSHBG, age, ratio of family income to poverty; total energy, Ln(iron) and Ln(zinc) intake on the first day; fish eaten during the past 30 days, moderate recreational activities, body mass index, hematocrit, serum continine, Ln(serum albumin), Ln(serum copper), Ln(serum zinc) were adjusted.

## 4 Discussion

Lead exposure may be associated with hormonal changes, particularly in children, but there have been few studies on the health impact of metal exposure among children and adolescents (21). Our results showed an overall positive trend between BLL and E2 in males aged 8-11 years and 16-19 years. This association in the former age group was consistent with the findings of Mohamed A.M. Khalaf et al. (29). The mechanism may be that blood lead disrupts the balance of sex hormones in the hypothalamic-pituitary-gonadal system, especially at the hypothalamic level (29). In contrast, we found a significant trend of decreasing E2 in females for each elevated BLL after stratification for sex, consistent with other reports (30). Interestingly, we found an inverted U-shaped association between BLL and E2 with the point of inflection at 1.86 μg/L in female adolescents aged 16-19 years. That is, a low BLL promoted elevated levels of E2 while an excessive BLL caused a decrease in E2. There are two possible mechanisms by which a low BLL can promote an elevated E2 level. First, animal experiments found that a low BLL promoted a significant increase in gonadotropin-releasing hormone mRNA, which may stimulate the secretion of Follicle-Stimulating Hormone (FSH) and Luteinizing Hormone (LH), thereby causing an increase in the E2 levels (31). Second, blood lead increases homocysteine concentrations (32). Homocysteine is an N-methyl-D-aspartate agonist (33) that stimulates FSH and LH release (34). The reason for the decrease in E2 caused by high BLL may be the inhibition of steroid synthesis at the ovarian level. In an animal experiment of female rats, Prakash Pillai et al. found that lead could inhibit ovarian steroidogenesis by downregulating steroidogenic acute regulatory protein (StAR) expression, inhibiting ovarian steroidogenic enzymes, and increasing lipid peroxidation, leading to a decrease in serum E2 levels (35). Another possible mechanism is that lead could affect the expression of cyclin B1, a peptide related to ovarian granulosa cell proliferation and induce the expression of caspace-3, an apoptosis-related peptide, ultimately increasing the percentage of apoptosis in ovarian granulosa cells (36). Our findings differed from a study by Pollack who found that lead was positively with E2 (14). They enrolled 252 premenopausal women in Buffalo, New York and examined the associations of lead, cadmium, and mercury with reproductive hormones. The inconsistent results could be because their study population was premenopausal women, while we evaluated children. Further large-scale and longitudinal studies are needed to confirm this. E deficiency will result in bone demineralization, and the lead in the bone will be released into the blood in advanced bone demineralization (37). This can ultimately cause a vicious cycle, and thus, E2 suppression by BLL is important.

The results of our study showed that LnSHBG significantly increased with LnBLL in the Q4 range compared to that in the lowest LnBLL quartile group in male participants of all ages. Meanwhile, there was a significant positive correlation between BLL and SHBG in females, especially when the LnBLL quartiles are between Q3 and Q4. In addition, we found a U-shaped relationship between BLL and SHBG with an inflection point of 1.86 μg/L in female adolescents aged 16-19 years, indicating an inverse relationship between BLL and SHBG when BLL was <1.86 μg/L, and lead overload could lead to reduced SHBG. Previous studies have shown that excessive lead exposure can lead to hepatotoxicity by inducing oxidative injury and inflammation (38). Lead and other toxic metals have pro-oxidant and endocrine-disrupting properties, and studies have demonstrated that lead exposure may interfere with thyroid function by accumulating in the thyroid gland or affecting its regulation (39, 40). The production and maturation of thyroid hormones can increase blood SHBG levels (41). Thus, high BLL may indirectly affect the blood SHBG levels. These factors mentioned above may partially explain the negative correlation between them. Nevertheless, the mechanisms involved in the elevation of SHBG if the BLL is >1.86 μg/L have not been elucidated.

Our findings differed from a study reporting no relationship of blood lead with E2 and SHBG in men aged 50-75 years by Rotter et al. (42). They evaluated 313 men aged 50-75 years, and performed enzyme-linked immunosorbent assay to determine the concentrations of SHBG, E2, free testosterone (FT), and TT. Meanwhile, our study population comprised 8-19-year-old children and adolescents.

## 5 Limitations

To the best of our knowledge, this is the first study of relatively large sample size to assess the association of blood lead with E2 and SHBG in 8-19-year-old children and adolescents. Our findings provide prospective evidence for future clinical studies. We found U-shape and inverted U-shape patterns for blood lead and E2 with SHBG respectively for the first time. Moreover, we performed a subgroup analysis and found the inflection point for BLL to be 1.86 μg/L, which is lower than the safe reference value of 3.5 µg/dL set by the Centers for Disease Control (CDC) for blood lead in children (43). However, this study also has some limitations. First, there are restrictions on real-world research such as the data are often incomplete, inaccurate and biased. Second, our study was limited to children and adolescents, and the age stratification could be more detailed to identify sensitive age groups. Third, age at menarche is considered one of the indicators of estrogen exposure, and some studies have shown that the later the age at menarche, the lower the E2 level (44). The use of estrogen products may also have an impact on the result of serum E2 level. However, these factors could not be included as covariates in the analysis because of insufficient data from NHANES 2013-2016, making it difficult for us to assess their impact on the results obtained. Fourth, although SHBG is recognized to regulate estradiol transport and bioavailability, and the BLL values at the turning points of the two curves are almost equal, whether lead affects the affinity of SHBG for E2 in the circulation and affects the binding of the two has rarely been reported, and the mechanism still remains unclear and warrants further exploration (45).

## 6 Conclusions

This study found an inverted U-shaped association of BLL with E2 and a U-shaped association between BLL and SHBG in female adolescents aged 16-19 years. This indicates that adjusting blood lead exposure to mitigate the effects of lead on growth and development is important for adolescents aged 16-19 years. Controlling the BLL below 1.86 μg/L may minimize the damage to E2. Meanwhile, the optimal BLL to eliminate its effect on SHBG is still unclear. The almost coincidental overlap of BLL values at the turning points of the two curves is worthy of further investigation to determine the optimal level of blood lead.

## Data availability statement

The original contributions presented in the study are included in the article/**Supplementary Material**. Further inquiries can be directed to the corresponding author.

## Author contributions

KP and CZ contributed to conception and design of the study. YH extracted the data and organized the database. ZC and CZ performed the statistical analysis. KP wrote the first draft of the manuscript. CZ, RT, ZC, and YH wrote sections of the manuscript. All authors contributed to to the article and approved the submitted version.
