# Supplementary material for: Association of blood lead with estradiol and sex hormone-binding globulin in 8-19-year-old children and adolescents
Source: Front Endocrinol (Lausanne). 2023 Feb 8;14:1096659. doi: 10.3389/fendo.2023.1096659 (PMC9944751; doi:10.3389/fendo.2023.1096659)
Supplement: Supplementary file 1 [file Table_1.doc]

**Supplementary table 1**

Association between LnBLL (μmol/L) and LnE2 (pg/mL)

|  | **Model 1 β (95% CI)** | **Model 2 β (95% CI)** | **Model 3 β (95% CI)** |
| --- | --- | --- | --- |
| Blood lead level (μmol/L) | -4.8128 (-7.0114, -2.6142) ^***^ | -0.0804 (-1.3502, 1.1893) | 0.065260 (-1.2293, 1.3598) |
| Stratified by sex |  |  |  |
| Male | 2.3928 (0.0150, 4.7706) ^*^ | 0.5255 (-0.5075, 1.5585) | 1.0248 (-0.0288, 2.0783) |
| LnBLL (Quartile) |  |  |  |
| Q1 | Reference | Reference | Reference |
| Q2 | 0.0688 (-0.1417, 0.2793) | 0.0619 (-0.0297, 0.1535) | 0.0802, (-0.01259, 0.1730) |
| Q3 | -0.0720 (-0.2797, 0.1356) | 0.0499 (-0.0400, 0.1398) | 0.0720, (-0.0205, 0.1645) |
| Q4 | -0.0438 (-0.2482, 0.1607) | 0.0537 (-0.0356, 0.1431) | 0.0806, (-0.0141, 0.1754) |
| P for trend | 0.019 | 0.048 | <0.001 |
| Female | -5.5305 (-8.8724, -2.1886) ^**^ | -1.0087 (-3.2181, 1.2007) | -1.1313 (-3.3291, 1.0666) |
| LnBLL (Quartile) |  |  |  |
| Q1 | Reference | Reference | Reference |
| Q2 | -0.2815 (-0.4986, -0.0644) ^*^ | -0.0207 (-0.1664, 0.1250) | -0.0266, (-0.1742, 0.1210) |
| Q3 | -0.6009 (-0.8502, -0.3515) ^***^ | -0.0995 (-0.2681, 0.0690) | -0.0954, (-0.2671, 0.0762) |
| Q4 | -0.9092 (-1.1839, -0.6345) ^***^ | -0.1203 (-0.3097, 0.0691) | -0.1153, (-0.3068, 0.0761) |
| P for trend | <0.001 | <0.001 | <0.001 |
| Stratified by race/ethnicity |  |  |  |
| Non-Hispanic White | -4.2443 (-8.8107, 0.3221) | 1.0384 (-1.7414, 3.8183) | 1.1641, (-1.7941, 4.1223) |
| Non-Hispanic Black | -21.6820 (-27.9767, -15.3873) ^***^ | -2.6691 (-6.6886, 1.3504) | -2.7115 (-6.9047, 1.4817) |
| Mexican American | -8.4346 (-13.3870, -3.4822) ^***^ | -1.7884 (-4.3803, 0.8036) | -2.065140 (-4.7230, 0.5928) |
| Other race/ethnicity | -0.8525 (-3.9201, 2.2151) | 0.0439 (-1.7001, 1.7878) | 0.1848 (-1.5797, 1.9494) |

Model 1: no covariates were adjusted

Model 2: age, sex, and race/ethnicity were adjusted

Model 3: age, sex, race/ethnicity, ratio of family income to poverty; total energy, cholesterol, Ln(iron) and Ln(zinc) intake on the first day; fish eaten during the past 30 days, moderate recreational activities, body mass index, Ln(serum copper) were adjusted

*Abbreviation*: BLL Blood lead levels; E2 Estradiol

^*^ *P*  < 0.05，^**^ *P*  < 0.01，^***^ *P*  < 0.001
