# Supplementary material for: Association of blood lead with estradiol and sex hormone-binding globulin in 8-19-year-old children and adolescents
Source: Front Endocrinol (Lausanne). 2023 Feb 8;14:1096659. doi: 10.3389/fendo.2023.1096659 (PMC9944751; doi:10.3389/fendo.2023.1096659)
Supplement: Supplementary file 2 [file Table_2.doc]

**Supplementary table 2**

Association between LnBLL (μmol/L) and LnSHBG (pg/mL)

|  | **Model 1 β (95% CI)** | **Model 2 β (95% CI)** | **Model 3 β (95% CI)** |
| --- | --- | --- | --- |
| Blood lead level (μmol/L) | 0.9891 (-0.0388, 2.0170) | 0.9599 (0.0514, 1.8683) ^*^ | -0.0733 (-0.8372, 0.6905) |
| Stratified by sex |  |  |  |
| Male | 0.1319 (0.0616, 0.2022) ^***^ | 0.1176 (0.0651, 0.1701) ^***^ | 0.0408 (-0.0044, 0.0860) |
| LnBLL (Quartile) |  |  |  |
| Q1 | Reference | Reference | Reference |
| Q2 | 0.0757 (-0.0538, 0.2051) | 0.0380 (-0.0594, 0.1354) | -0.0328 (-0.1122, 0.0466) |
| Q3 | 0.1665 (0.0388, 0.2942) ^*^ | 0.0846 (-0.0110, 0.1802) | -0.0090 (-0.0876, 0.0697) |
| Q4 | 0.2852 (0.1595, 0.4110) ^***^ | 0.1839 (0.0889, 0.2789) ^***^ | 0.0605 (-0.0190, 0.1400) |
| P for trend | <0.001 | <0.001 | <0.001 |
| Female | 0.1121 (0.0410, 0.1832) ^***^ | 0.0697 (0.0000, 0.1394) | 0.0484 (-0.0089, 0.1058) |
| LnBLL (Quartile) |  |  |  |
| Q1 | Reference | Reference | Reference |
| Q2 | -0.0289 (-0.1242, 0.0663) | -0.0575 (-0.1484, 0.0334) | 0.0009 (-0.0739, 0.0757) |
| Q3 | 0.1589 (0.0496, 0.2683) ^**^ | 0.1107 (0.0056, 0.2159) ^*^ | 0.1044 (0.0154, 0.1934) ^*^ |
| Q4 | 0.2253 (0.1048, 0.3458) ^***^ | 0.1650 (0.0469, 0.2832) ^**^ | 0.1171 (0.0209, 0.2133) ^*^ |
| P for trend | <0.001 | <0.001 | <0.001 |
| Stratified by race/ethnicity |  |  |  |
| Non-Hispanic White | -0.1067 (-2.2095, 1.9960) | 0.9343 (-1.0330, 2.9017) | -0.0125 (-1.6993, 1.6742) |
| Non-Hispanic Black | 6.4539 (3.8147, 9.0931) ^***^ | 2.4938 (-0.0057, 4.9933) | 2.0384 (-0.1782, 4.2550) |
| Mexican American | 2.8477 (0.5573, 5.1381) ^*^ | 2.2597 (0.1842, 4.3352) ^*^ | 0.9282 (-0.8867, 2.7432) |
| Other race/ethnicity | 0.3048 (-1.1181, 1.7277) | 0.3014 (-0.9970, 1.5997) | -0.4504 (-2.4957, 1.5948) |

Model 1: no covariates were adjusted

Model 2: age, sex, and race/ethnicity were adjusted

Model3: age, sex, race/ethnicity, ratio of family income to poverty; total energy, Ln(iron) and Ln(zinc) intake on the first day; fish eaten during the past 30 days, moderate recreational activities, body mass index, hematocrit, serum continine, Ln(serum albumin), Ln(serum copper), Ln(serum zinc) were adjusted

*Abbreviation*: BLL Blood lead levels; E2 Estradiol; SHBG Sex hormone-binding globulin

^*^ *P*  < 0.05，^**^ *P*  < 0.01，^***^ *P*  < 0.001
